# Characterization of ciprofloxacin-resistant and ESBL-producing *Salmonella enteric* serotype Derby in Eastern China

**DOI:** 10.1186/s12866-019-1434-6

**Published:** 2019-03-18

**Authors:** Zhaojun Xu, Qifa Song, Chunhua Li, Yefei Zhan

**Affiliations:** 1 Intensive Care Unit, Ningbo No. 2 Hospital, Zhejiang, Zhejiang Province People’s Republic of China; 2 Department of Microbiology, Ningbo Municipal Centre for Disease Control and Prevention, No. 237 Yongfeng Road, Haishu District, Ningbo, 315010 Zhejiang Province People’s Republic of China

**Keywords:** *Salmonella* Derby, ciprofloxacin resistance, extended-spectrum β-lactamase (ESBL)-production, pulsed-field gel electrophoresis (PFGE), mechanism of antibiotic resistance

## Abstract

**Background:**

Fluoroquinolone resistance and ESBL-production are concurrently found in a limited number of *Salmonella* serotypes. The present study was aimed to characterize fluoroquinolone-resistant and ESBL-producing *Salmonella enteric* serotype Derby (*S*. Derby) isolates in terms of antimicrobial susceptibility, relevant genetic mechanisms, and PFGE.

**Results:**

From 2013 to 2017 in Ningbo China, 52 *S.* Derby isolates were identified out of 826 non-typhoidal *Salmonella* isolates from patient feces, food, and environmental water samples. Three *S.* derby isolates were identified to be fluoroquinolone-resistant and ESBL-producing with cefotaxime MIC of 64 μg/mL and ciprofloxacin MIC of 4 μg/mL. The three isolates contained the same genetic structure of quinolone resistance, including a silent *gyrA* mutation S (TCC) 83S (TCT) and three PMQR genes *qnrB*, *qnrS* and *aac(6′)-Ib-cr*. As withβ-lactams resistance mechanisms, two isolates contained *bla*_TEM_, *bla*_OXA_, and *bla*_CTX-M_ genes and one isolate contained *bla*_OXA_ and *bla*_CTX-M_ genes. Additionally, two isolates displayed more identical PFGE pattern than the third isolate, whereas three isolates showed the same plasmid profile of I1, W and P by PCR-based replicon typing*.* The conjugation experiment showed no dissemination of β-lactam resistance by direct contact among isolates; the transformation experiment failed to transfer plasmid conferring ampicillin resistance to *E. coli* DH5a.

**Conclusion:**

The present study demonstrates the emerging fluoroquinolone-resistant and ESBL-producing *S.* Derby in both humans and the environment. Seeing that *S.* Derby has become one of the most common *Salmonella* serotypes, this situation gives rise to a new major risk of food-borne diseases.

## Background

Non-typhoidal *Salmonella* species that contaminate food products of animal origin are among the leading causes of human foodborne diseases globally [1, 2]. Typically, most cases of salmonellosis are gastroenteritis that is not complicated and does not require antimicrobials for treatment, but severely invasive salmonellosis can sometimes be found in infants, the elderly and patients with weakened immunity, which needs appropriate treatment with antimicrobials such as fluoroquinolones and β-lactams. As fluoroquinolones and β-lactams are now recommended for salmonellosis treatment, resistance to these drugs in *Salmonella* species can lead to failure to cure salmonellosis and subsequent serious clinical outcomes. To date, both our work on *Salmonella* resistance and previous literature have proved that concurrent fluoroquinolone resistance and extended-spectrum β-lactamase (ESBL)-production are found only in a limited number of *Salmonella* serotypes, such as Typhimurium, Indiana and Kentucky [3, 4].

*Salmonella* enteric serotype Derby (*S.* Derby) is one of the numerous non-typhoidal *Salmonella* serotypes and has been recognized as a food-borne pathogen. Previous studies have showed that *S.* Derby is mainly associated with pig origin in many parts of the world [5]. Recently, *S.* Derby, which was frequently resistant to tetracycline and sulfisoxazole, was found to become one of the most common *Salmonella* serotypes in diarrheal patients in China [6].

In the present study, we first identified several *S.* Derby isolates that were highly resistant to fluoroquinolones and β-lactams, especially to be ESBL-producing. This situation rendered the predominant *S.* Derby as a new great threat to human health because of the very limited choices of antibiotics for treatment of infection by highly fluoroquinolone-resistant and ESBL-producing *Salmonella* species. The present study was undertaken to characterize the mechanisms of antimicrobial resistance and distribution patterns of the fluoroquinolone-resistant and ESBL-producing *S.* Derby.

## Methods

### Bacterial isolates

A surveillance project for *Salmonella* species has been carried out since 2005 in the study city Ningbo, Zhejiang province, China. Three categories, namely stool, food and river water, of samples were included. *Salmonella* isolation was performed as follows. Briefly, stool specimens were first put in tetrathionate brilliant-green broth or selenite broth to enrich, followed by cultivation on *Salmonella-Shigella* agar, or CHROMagar *Salmonella* agar (bioMerieux, Paris, France). Then, food samples were treated sequentially through enrichment with buffered peptone water (Difco, USA), selenite cystine (Difco) and broth tetrathionate (Difco) broth and subsequent selective cultivation with CHROMagar *Salmonella* agar. Environmental water samples were filtered using membranes of 0.45 μm pore size (Merck Millipore, USA), and the membranes were cut into pieces and treated with similar enrichment and subsequent selective cultivation as food samples [7]. Colonies with typical *Salmonella* appearance were tested for biochemical reactions by API20E identification system (bioMerieux, Paris, France). O and H antigens were determined by slide agglutination using *Salmonella*-specific antisera (Denka Seiken, Japan). Isolates from food and water samples were described altogether as an environmental source.

### Antimicrobial susceptibility testing (AST)

All *S.* Derby isolates were tested for susceptibility to nine commonly administered antibiotics using the Kirby-Bauer disc diffusion method specified in the Clinical and Laboratory Standards Institute (CLSI) document M02-A12 [8]. The antibiotic discs (Oxoid, Hampshire, England) included ampicillin (10 μg), chloramphenicol (30 μg), tetracycline (30 μg), ciprofloxacin (5 μg), nalidixic acid (30 μg), gentamicin (10 μg), cefotaxime (30 μg), meropenem (10 μg) and sulfamethoxazole/trimethoprim (1.25/23.75 μg). AST results were interpreted according to the guidelines in the CLSI M100-S27 [9]. ESBL-producing *S.* Derby isolates were confirmed using the test specified in the CLSI document M02-A12, namely, a ≥ 5-mm increase in the zone diameter for cefotaxime-clavulanate compared with the zone diameter for cefotaxime was regarded as being ESBL-producing. *Escherichia coli* ATCC 25922 was used for quality control. Minimal inhibitory concentrations (MICs) of cefotaxime and ciprofloxacin for the fluoroquinolone-resistant and ESBL-producing *S.* Derby isolates were determined using the E-test strips (Oxoid, Hampshire, England).

### Detection of antimicrobial resistance-relevant genetic determinants

Three β-lactamase genes *bla*_TEM_, *bla*_OXA_, and *bla*_CTX-M_ were detected using PCR primers as described previously [10, 11]. To analyze genetic mechanisms for fluoroquinolone resistance, *gyrA*, *qnrB* and *ParC* genes in the quinolone resistance-determining region (QRDR) were amplified and sequenced in all isolates using primers as previously described [12]. To determine *gyrA* mutations, the sequenced *gyrA* amplicons were aligned against the corresponding *gyrA* sequence of *Salmonella* Typhimurium LT2 using the CLUSTAL W software (European Bioinformatics Institute). We also detected the transferable plasmid-mediated quinolone resistance (PMQR) genes that typically include pentapeptide repeat protein-encoding *qnrA*, *qnrB* and *qnrS*, an aminoglycoside acetyltransferase-encoding enzyme variant *aac(6′)-Ib-cr* and an efflux-pump-encoding *qepA* [13]. In addition, to assess the transmissible elements of resistance, class 1 and class 2 integrons were amplified using the method based on previous literature [14].

### Pulsed-field gel electrophoresis (PFGE)

All *S.* Derby isolates were subtyped by PFGE using the restriction enzyme *XbaI* following the PulseNet standardized protocol [14, 15]. *S.* Braenderup (H9812) was used as a reference strain. Clonal relatedness was analyzed by BioNumerics software (Applied Maths, Sint-Martens-Latum, Belgium). Cluster analysis was performed based on the unweighted pair-group method with 1.5% optimization and 1.0% band matching tolerance.

### Plasmid profile and conjugation experiment

The fluoroquinolone-resistant and ESBL-producing *S.* Derby isolates were inoculated into 200 mL of Luria-Bertani (LB) broth and cultivated for 12 h with shaking of 150 rpm. Then, the plasmids in 200 mL of broth were extracted by a large-volume preparation method using commercial EasyPure Hipure Plasmid Maxiprep Kit (Transgen, Beijing, China). Plasmid profiles were obtained for each isolate using electrophoresis. A PCR-based plasmid typing method was performed to trace the drug-conferring plasmids using 18 pairs of primers representing FIA, FIB, FIC, HI1, HI2, I1-Ig, L/M, N, P, W, T, A/C, K, B/O, X, Y, F, and FIIA plasmid replicons [16].

Plasmid transformation into *E. coli* DH5a (TaKaRa Biotechnology, Dalian, China) was conducted for the extracted plasmids to assess β-lactam resistance dissemmination by plasmid. Transformants were selected on LB agar containing 50 μg/mL ampicillin. The highly resistant *S.* Derby isolates were also tested for resistance transmission ability among isolates through conjugation experiment with rifampin-resistant *E. coli* c600 as recipients according to previous literature [17]. Transconjugants were selected on MacConkey agar plates containing ceftriaxone (16 μg/mL) and rifampin (50 μg/mL).

## Results

### Bacterial isolation

The first *S.* Derby isolate was identified in 2013. From 2013 to 2017, 826 non-typhoidal *Salmonella* isolates and 52 (6%) *S.* Derby isolate were collected. The top three most common *Salmonella* serotypes were *S*. Typhimurium (242 isolates, 29%), *S*. Enteritidis (95 isolates, 12%) and *S.* Derby. Of the 52 *S.* Derby isolates, 30 were isolated from faeces of 2200 diarrheal patients each year who visited hospitals in Ningbo, China and 22 from environmental sources (10 from food samples comprised of 570 meat samples and 80 egg samples; 12 from 115 river water samples that were taken from rivers during 2013–2017 as previously reported [7] (Table 1). All diarrheal patients were out-patients complaining of acute diarrhoea and fully recovered within 1**–**5 days.

**Table 1:** Antimicrobial susceptibility testing results for *S.* derby isolates from different sources

Antimicrobial agent	No. of resistant isolates			*P*
	Environmental source (*N* = 22)	Patient source (*N* = 30)	Total (*N* = 52)	
Ampicillin	9 (41%)	19 (63%)	28 (54%)	0.37
Cefotaxime	1 (5%)	3 (10%)	4 (8%)	–
ESBL	1 (5%)	3 (10%)	4 (8%)	–
Nalidixic acid	16 (73%)	28 (93%)	44 (85%)	0.55
Ciprofloxacin	4 (18%)	6 (20%)	10 (19%)	–
Gentamicin	3 (14%)	3 (10%)	6 (11%)	–
Chloramphenicol	10 (45%)	19 (63%)	29 (56%)	0.43
Tetracycline	15 (68%)	20 (67%)	35 (67%)	0.96
Trimethoprim-sulfamethoxazole	18 (82%)	22 (73%)	40 (77%)	0.80
MDR*	7 (32%)	18 (60%)	25 (48%)	0.22
Total events	84 (38%)	141 (47%)	225 (44%)	0.04

*Abbreviation: *MDR* multidrug resistant, refers to concurrent resistance to ampicillin, chloramphenicol, and trimethoprim-sulfamethoxazole

### AST results

As with the AST results for *S.* Derby isolates, only eight isolates were susceptible to all antimicrobials, whereas 44 (85%) isolates were resistant to at least one antimicrobial agent (Table 1). Resistance to nalidixic acid (85%), sulfamethoxazole/trimethoprim (77%), tetracycline (67%), chloramphenicol (56%) and ampicillin (54%) was among the top three highest levels. Resistance to ciprofloxacin (19%), gentamicin (11%), cefotaxime (8%), and ESBL (8%) was at a relatively low level. Moreover, two isolates (designated as Salm1125 and Salm1165) from feces of diarrheal patients and one (designated as Salm1184) from river water were identified to be both fluoroquinolone-resistant and ESBL-producing. No significant difference for each antimicrobial agent was observed between patient and environment samples. However, taking the resistance events as a whole, the occurrence rate of total resistance events in the patient isolates was significantly higher than the corresponding rate in the environmental isolates by Pearson chi-square test (*P* = 0.04).

### Analysis of three highly antimicrobial-resistant *S.* Derby isolates

**Fig. 1:**
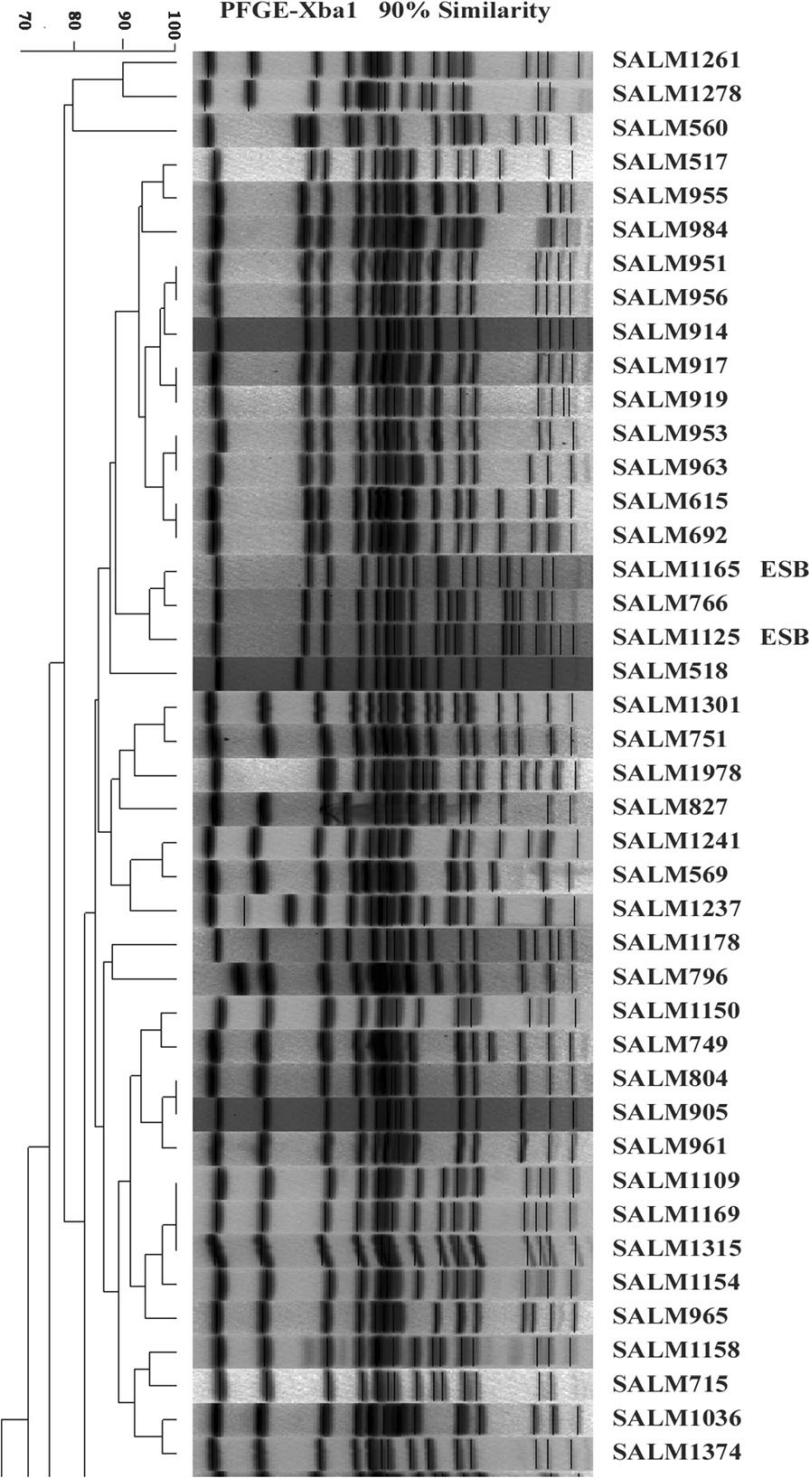
PFGE patterns of 52 *S.* Derby isolates with three highly antibiotic-resistant *S.* Derby isolates (Salm1125, Salm1165, and Salm1184), showing overall moderate similarity in PFGE patterns

**Fig. 2:**
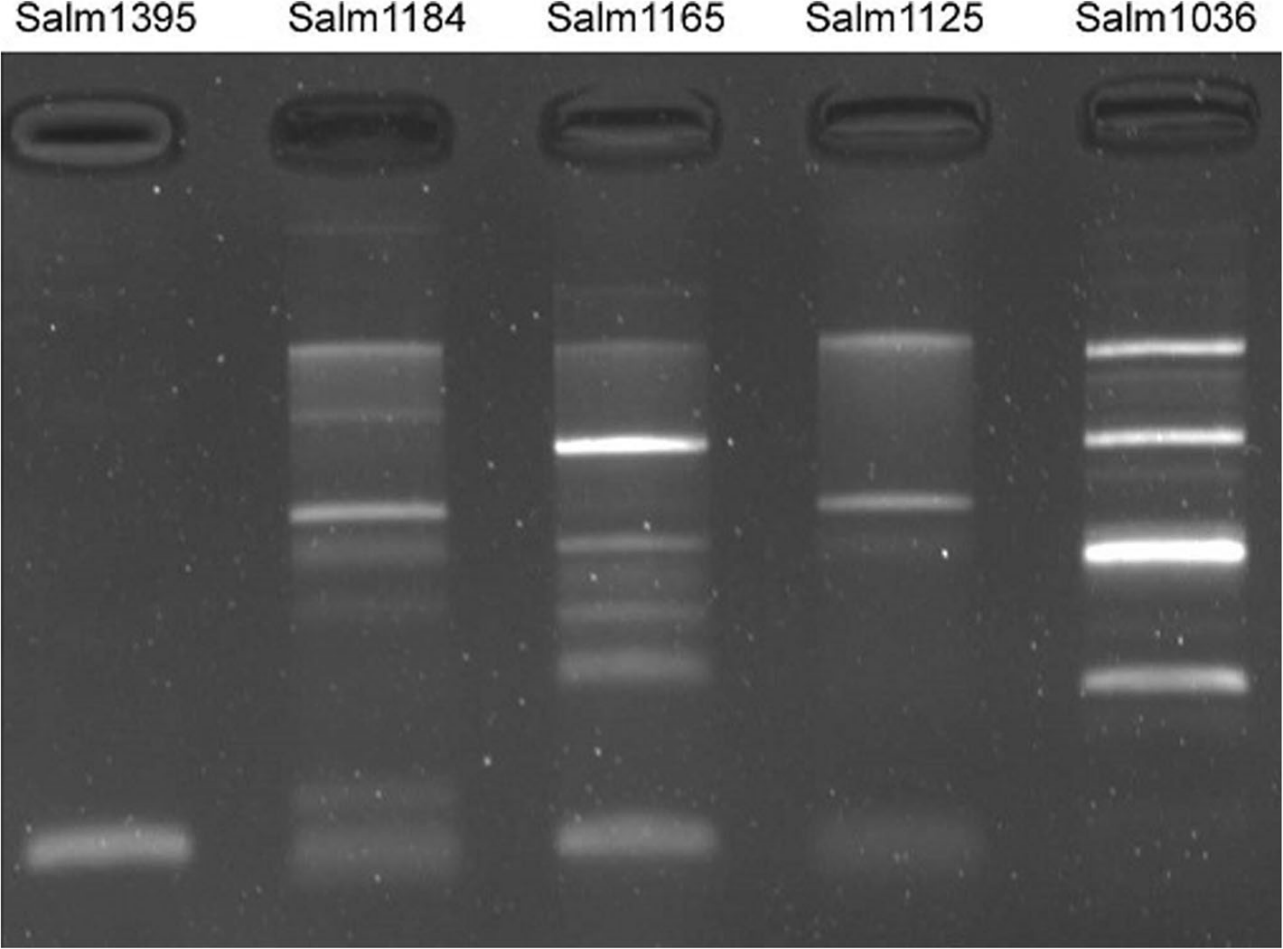
Plasmid profiles of three highly antibiotic-resistant *S.* Derby isolates (Salm1125, Salm1165 and Salm1184) and two pan-sensitive *S.* Derby isolate (Salm1036 and Salm1395), showing complex plasmid composition

Seeing that concurrently fluoroquinolone-resistant and ESBL-producing *S.* Derby isolates are clinically important, three such *S.* Derby isolates were further analyzed. Two isolates were isolated from patients who showed no epidemiological relationship and had five**–**six liquid stools per day. The two patients were fully recovered in 3 days with orally administered medications. All the three isolates showed the same pan-resistance profile to nine antimicrobial agents tested, namely, resistant to all agents. Their MIC results were cefotaxime MIC of 64 μg/ml and ciprofloxacin MIC of 4 μg/ml. In these isolates, same genetic structures for quinolone resistance were detected, including a silent *gyrA* mutation S (TCC) 83S (TCT) encoding the same amino acid despite the mutation and three PMQR genes *qnrB*, *qnrS* and *aac(6′)-Ib-cr*. Regarding β-lactam resistance genes, Salm1125 and Salm1165 harbored three types of relevant genes *bla*_TEM_, *bla*_CTX-M_, and *bla*_OXA_, whereas Salm1184 harbored two types of genes *bla*_CTX-M_ and *bla*_OXA_. Blasting showed that the sequences for each *bla*_TEM_, *bla*_CTX-M_ and *bla*_OXA_ were the same. The NCBI accession numbers were MK296540 (*bla*_OXA-1_), MK296541 (*bla*_CTX-M-55_), and MK296542 (*bla*_TEM-1_). All isolates contained *int1* gene with complete structure. Each isolate showed different PFGE patterns and could be distinguished from the pan-sensitive isolate (Salm1395) (Fig. 1). Salm1125 and Salm1165 of patient sources displayed more genetic similarity with each other, whereas Salm1184 from river water showed a greater difference from Salm1125 and Salm1165. Three isolates displayed the identical multiple-plasmid replicon typing profile of HI1, W and P, suggesting multiple plasmids in each isolate. The electrophoresis profile also demonstrated complex plasmid pattern (Fig. 2). The conjugation experiment was not able to generate antibiotic-resistant transconjugants on MacConkey agar plate. No plasmid that conferred ampicillin resistance was transferred into *E. coli* DH5a by transformation experiment.

## Discussion

*S.* Derby was initially found to infect animals, especially pigs and turkeys [18]. A previous study in France dealing with 196 *S.* Derby isolates proved that *S.* Derby was among the second most found *Salmonella* serotypes from pigs [19]. In recent years, public concern about *S.* Derby arose owing to its increasing importance in food-borne diseases in humans. In China, *S.* Derby was reported to be among the three most common serotypes of non-typhoidal *Salmonella* isolated from patients with diarrhoea [6].

In addition to the high prevalence of *S.* Derby in both humans and several types of animals, the current study proved that the overall prevalence of antibiotic resistance among *S.* Derby isolates was at a high level in terms of resistance rates to ampicillin, tetracycline, chloramphenicol, and sulfamethoxazole/trimethoprim. These results are consistent with those in a previous report [6]. The isolates from patients were found to be at a higher level of antimicrobial resistance than the environmental isolates. Moreover, this study first identified both ciprofloxacin-resistant and ESBL-producing *S.* Derby. Fluoroquinolone, one of which is ciprofloxacin, and third-generation cephalosporin are often used to treat salmonellosis. Previous literature has showed that *Salmonella* species resistant to both types of drugs are not very common as a whole. Our previous study concerning the overall fluoroquinolone resistance rate exhibited such rate to be about 1% [3]. To date, relatively few *Salmonella* species with concurrent resistance were reported, mainly *S.* Kentucky, *S. Indiana*, *S.* Typhimurium, and *S.* Enteritidis. Consequently, the appearance of ciprofloxacin-resistant and ESBL-producing *S.* Derby adds another type of highly antibiotic-resistant *Salmonella* species. Due to the limited number of resistance events for each drug, no significant difference was observed between patient and environment samples. However, taking the resistance events as a whole, a significantly higher occurrence rate of total resistance events was in the patient isolates than in the environmental isolates (*P* = 0.04).

As with the three highly antibiotic-resistant *S.* Derby isolates, cefotaxime MIC was 64 μg/mL, which is similar to cefotaxime MIC in other *Salmonella* species. Ciprofloxacin MIC value was 4 μg/mL, which is close to the MIC values in most ciprofloxacin-resistant *Salmonella* strains and lower than ciprofloxacin MIC value in *S.* Kentucky and *S. Indiana* (16 and > 32 μg/mL) [3]. When analyzing molecular mechanisms of ciprofloxacin resistance in the three *S.* Derby isolates, we found silent *gyrA* mutation S (TCC) 83S (TCT), namely, encoding the same amino acid despite the one-base mutation. This finding contrasts with the point of view that *gyrA* mutations are the major genetic structures causing quinolone resistance [20]. Additionally, three PMQR genes *qnrB*, *qnrS* and *aac(6′)-Ib-cr* were also detected in these isolates. The previous study has proved that *qnrB* is a more common indicator of fluoroquinolone resistance because it is only found in all ciprofloxacin-resistant *Salmonella* isolates, by contrast, *gyrA* mutations are not necessarily the cause of such resistance because *gyrA* mutations are often found in reduced ciprofloxacin-susceptible strains and absent in a number of ciprofloxacin-resistant strains [3]. The three *S.* Derby isolates all contained more than one type of ESBL-relevant genes. Salm1125 and Salm1165 contained *bla*_CTX-M_, *bla*_OXA_, and *bla*_TEM_, and Salm1184 contained *bla*_CTX-M_ and *bla*_OXA_. The *bla*_CTX-M_, *bla*_OXA_ and *bla*_TEM_ genes are three most encountered β-lactam resistance genes, which may confer ESBL production in Enterobacteriaceae [21]. Although β-lactam-relevant genes have been detected in several *Salmonella* serotypes [22], rarely there are more than one types existing simultaneously in one strain as our findings. Especially, all the isolates contained *int1* gene with a complete structure, which enhances the ability of gene re-arrangement and antibiotic resistance spread among strains.

Finally, the three isolates displayed different PFGE patterns, suggesting that the isolates are genetically different. The electrophoresis profile of each isolate demonstrated multiple plasmids (Fig. 2). Three types of plasmid replicons HI1, W and P All were simultaneously detected in each of three isolates, indicating the complex plasmid composition. The three types of replicons were previously found in *S.* Typhi and *S.* Typhimurium [16]. The conjugation experiment in the present study showed that β-lactam resistance was not able to spread among isolates. The transformation experiment was also unable to transfer plasmid conferring ampicillin resistance into *E. coli* DH5a.

## Conclusion

The present study demonstrates that highly antibiotic-resistant *S.* Derby with fluoroquinolone resistance and ESBL production has been emerging. Even worse, *S.* Derby has become one of the most common *Salmonella* serotypes and causes illness in both humans and animals. This situation gives rise to a new looming risk of food-borne diseases by *S.* Derby.

## Abbreviations

AST: Antimicrobial susceptibility testing
ESBL: extended-spectrum β-lactamase
LB: Luria-Bertani broth
MIC: minimal inhibitory concentration
PFGE: pulsed-field gel electrophoresis
PMQR: transferable plasmid-mediated quinolone resistance
QRDR: quinolone resistance-determining region
S. Derby: Salmonella enteric serotype Derby
